# Teachers or Psychologists: Who Should Facilitate Depression Prevention Programs in Schools?

**DOI:** 10.3390/ijerph110505294

**Published:** 2014-05-15

**Authors:** Melanie S. Wahl, Jill L. Adelson, Margarete A. Patak, Patrick Pössel, Martin Hautzinger

**Affiliations:** 1 Department of Clinical Psychology and Psychotherapy, Eberhard Karls University, Schleichstrasse 4, Tübingen D-72076, Germany; E-Mails: margarete.patak@psycho.uni-tuebingen.de (M.A.P.); martin.hautzinger@uni-tuebingen.de (M.H.); 2 Department of Educational and Counseling Psychology, University of Louisville, Louisville, KY 40292, USA; E-Mails: jill.adelson@louisville.edu (J.L.A.); patrick.possel@louisville.edu (P.P.)

**Keywords:** school-based prevention, depression, qualification of group leaders, teachers, psychologists

## Abstract

The current study evaluates a depression prevention program for adolescents led by psychologists *vs.* teachers in comparison to a control. The universal school-based prevention program has shown its efficacy in several studies when implemented by psychologists. The current study compares the effects of the program as implemented by teachers *versus* that implemented by psychologists under real-life conditions. A total of 646 vocational track 8th grade students from Germany participated either in a universal prevention program, led by teachers (*n* = 207) or psychologists (*n* = 213), or a teaching-as-usual control condition (*n* = 226). The design includes baseline, post-intervention, and follow-up (at 6 and 12 months post-intervention). The cognitive-behavioral program includes 10 sessions held in a regular school setting in same-gender groups and is based on the social information-processing model of social competence. Positive intervention effects were found on the change in girls’ depressive symptoms up to 12 months after program delivery when the program was implemented by psychologists. No such effects were found on boys or when program was delivered by teachers. The prevention program can successfully be implemented for girls by psychologists. Further research is needed for explanations of these effects.

## 1. Introduction

Onset of major depression often occurs during adolescence [1] and is associated with increased risk of recurrent depressive episodes [2] and other psychopathology into adulthood [3]. Therefore, finding an effective intervention that prevents depression in adolescents is an important public health priority.

Prevention is considered one of the most effective strategies to reduce the burden associated with psychological diseases by the World Health Organisation [4]. However, little research has empirically examined the efficiency of prevention programs under real-life conditions. Only if the positive results of efficacy trials (lead under optimal conditions) can be proven to stand under real-life conditions in effectiveness trials (e.g., integrated into a classroom curriculum/lead by normal school personnel) is the widespread dissemination of such programs justified. The current study is the first that evaluates the effectiveness of a universal depression prevention program delivered by teachers compared to by psychologists under real-life conditions up to 12 months after program delivery.

### 1.1. Prevention of Depression

The Institute of Medicine’s [5] report on prevention of mental, emotional, and behavioral disorders presents clear evidence that major depression can be prevented. A recent meta-analytic review [6] revealed that 22% of depression incidents according to DSM can be prevented in adolescents independent from type of intervention. Cognitive-behavioral programs (CB) implemented by psychologists have shown positive prevention effects on depression scores in several studies. For example the Resourceful Adolescent Program (RAP; [7,8]), focusing on self-management and problem-solving, has shown significant improvements in depressive scores of adolescents compared to a control group up to 10 months after program delivery. A recent meta-analytic review showed that the Penn Resiliency Program (PRP), that teaches cognitive-behavioral and social problem-solving skills, significantly reduces depressive symptoms through at least 1-year post intervention [9]. The evaluation studies of the Problem Solving For Life Program (PSFL; [10]), with components of cognitive restructuring and problem-solving, found significant short-term effects on depressive scores of adolescents; however, those effects were not maintained over a follow-up period up to four years [10,11]. Finally the LARS & LISA program [12], described below, has also proven to be effective in several trials.

To overcome the burden associated with depressive disorders these results of effective prevention strategies are promising. Nevertheless the question remains how a widespread dissemination of these effective prevention programs can be organized. In addition, we do not know much about possible moderators (e.g., gender of participants, trainer’s professional background) of successful outcome of such a school-based, universal program.

Meta-analyses of studies aimed at preventing depression in children and adolescents have concluded that some efficacious interventions for the prevention or reduction of depressive symptoms in youth exist (e.g., [9,13,14]). On average, effect sizes (ES) of the various depression prevention programs have been small to modest. Moderators of these effects have included the type of sample (*i.e.*, universal, selective, indicated), participant attributes (e.g., age, gender, race), characteristics of the intervention (e.g., duration, content), interventionists (e.g., level of training), and timing of assessments (e.g., post-intervention, follow-ups of various lengths). The timing of assessments is relevant as effects of prevention in general and universal prevention in particular depends on the increase of depressive symptoms in the control group. Thus, it is not to be expected that prevention programs show effects on depressive symptoms in the short term (*i.e.*, post-intervention). Consistent with this expectation, a review found an increase in the magnitude of effect sizes of prevention programs on depressive symptoms at 6-month follow-up compared to baseline-post-intervention comparisons (for a review, see [15]). This hypothesis is also supported by a recent meta-analysis [14] that found no statistically significant effects of universal prevention programs on adolescent depression at post-intervention (*r* = 0.04, *p* = n.s.) but did find statistically significant effects at follow-up (*r* = 0.06, *p* < 0.001). Therefore we would not expect any effects to manifest immediately *after* the intervention took place. ES’s also differed depending on qualification of group leaders.

### 1.2. Qualification of Group Leaders

In school-based prevention programs of depression either psychologists or teachers are often used as group leaders. Both groups have their own advantages: psychologists are experts on psychological problems and their prevention and intervention [14] and have the possibility to start a new and unbiased relationship with the students as external group leaders without the necessity of performance appraisal. Teachers, on the other hand, can rely on their pedagogical background, know their group thoroughly, and can build on their long-term trustful relationship and an established set of rules, as well as conduct a kind of booster sessions by repeating the contents of the programs in appropriate situations later [16,17]. In regard to a widespread dissemination of prevention programs, teachers might be preferable because the programs can be implemented more easily in the normal school curricula, and for many schools it is too expensive to hire external psychologists.

There are no published studies comparing the effect of teachers and psychologist directly. Several programs have been evaluated either with teachers or with psychologists as group leaders and have shown positive outcomes with that specific type of group leader (e.g., [10,18,19,20]). Some programs have been implemented with teachers and with psychologists as group leaders but in separate studies. For example, the PRP [21,22] and the RAP [23,24] have shown consistent good effects when implemented by psychologists but much weaker (if any) effects when implemented by teachers. However, as these evaluations were conducted in different settings (e.g., primary care setting *vs.* school, schools with different socioeconomic background), with different participants (e.g., different age or gender ratio, elevated depressive symptoms or not), and often with slightly different ways of adoption of the program (e.g., including a parent module or not), it is difficult to compare the results, and therefore, no conclusion can be drawn in regard to the relative effect of teachers and psychologist as group leaders. For instance, in a meta-analysis of the effects of the RPR, Brunwasser, Gillham, and Kim [9] concluded that there was not enough power to distinguish between the effects of differently qualified group leaders. Although Sheffield *et al.*’s [25] evaluation of the effectiveness of the PSFL program included both teachers and psychologist as group leaders, the former conducted a universal and the latter an indicated program, so results are not comparable. In one study that did directly compare teachers and psychology graduate students, Shatté [26] found conflicting results regarding which leaders were more effect: although the teachers had a greater effect in the Penn Optimism Program (a cognitive behavioral antecedent of the PRP), the graduate students had a greater effect in a placebo program focusing on emotions. Both programs prevented depression compared to a non-treatment group.

Almost all meta-analyses reveal lower efficacy rates for teachers in comparison to psychologists as group leaders [14,15,27,28]. There are a smaller percentage of studies with significant reduction of depressive symptoms for programs being implemented by teachers (46%) than by others (for example psychology graduate students, mental health professionals, or members of the research team (58%) [27,28]. This is supported by a recent study that found no differences for post-intervention but clear differences concerning efficacy in the follow-up data: although teachers only achieved an average effect of *r* = 0.03 (*p* < 0.05, n = 11), the average effect for psychologists was considerably higher, *r* = 0.14 (*p* < 0.001, n = 38; Stice *et al.* [14]. Although Pössel, Schneider, and Seemann [15] also concluded in their review that psychologists seem to achieve better effects than teachers, they demanded systematic studies for this question. Only in Neil and Christensen’s [29] meta-analysis of nine Australian prevention programs did teachers achieve results as good as psychologists did, but the authors confounded improvement in depressive and anxiety symptoms so it is unclear how far this result applies for depression prevention programs only.

Altogether there is some evidence that teachers can implement depression prevention programs successfully, although the effect seems to be smaller than the effect of programs delivered by psychologists. According to Stice *et al.* [14], lower effect sizes of programs delivered by teachers may be due to teachers’ classroom responsibilities, less training and supervision, and, perhaps most importantly, that professional interventionists are able to refine their presentation strategies by repeated implementation. As no published study compares the effects of psychologist-led *vs.* teacher-led *depression* prevention programs directly, at this time no conclusion can be drawn regarding their differential results.

### 1.3. Gender Effect

Next to the influence of the type of group leader on the result, a moderating effect of characteristics of the participants is to be expected. Boys and girls of the same age are at different levels of development and show different levels of depressive symptoms; thus, in many epidemiological studies (e.g., [30,31,32]) female adolescents had up to twice as high of rates of depression compared to male adolescents and showed considerably more depressive symptoms.

Results of evaluation studies concerning gender effects in prevention are contradictory: some programs showed no gender effect (e.g., [7,19,24]), some demonstrated greater or even exclusive prevention effects for girls [32,33,34,35], and others found greater effects for boys [36,37]. Even studies of the same prevention program (e.g., PRP) found contrary results [22,26,38].

Meta-analyses show the same inconsistent picture in regard to gender effects. Stice *et al.* [14] found that studies with a greater ratio of girls had higher effect sizes than studies with more boys. In some meta-analyses, it depended on the timing of the evaluation; for instance, Horowitz and Garber [39] found greater results for girls at post-intervention but no gender effect in their follow-up data. Garber and Downs [40] found that studies indicate a gender effect at post-intervention but no gender differences at the follow-up, yet the effect size of studies with low and high percentage of females did not differ even at follow-up. Merry *et al.*’s [13] meta-analysis found inconsistent results: there were prevention effects in depressive disorder compared to a non-intervention group at post-intervention and three to nine months later for girls but only at post-intervention for boys; yet there were effects on depressive symptoms at post-intervention for both genders but only for boys three to nine months later. Finally, Passon *et al.* [27] found no gender differences for selective or indicative programs yet a higher rate of successful universal programs for boys (61%) than for girls (48%).

There is little consensus concerning the cause of these gender differences. A possible reason is the different baseline levels of depression of boys and girls, *i.e.*, higher depression scores for one sex may lead to more motivation to participate actively and to apply the new skills. In addition a higher baseline level allows for a greater reduction towards a non-clinical level [14,41]. Shatté [26] proposes different preferences of the genders for cognitive or social contents or, alternatively, different treatment by the group leader because of the dominant or disruptive behavior of the boys. Pössel *et al.* [35] suggest comorbid conduct problems as a possible reason for the smaller benefit of the boys. There is evidence against all of these interpretations. For example, controlling for baseline depression and conduct problems did not alter the gender effects in one study [22], and teaching girls in single-gender groups achieved the same effects as teaching them in co-ed groups in another study [38]. In the only available study explicitly addressing the gender question [35], neither conduct problems nor knowledge about contents of the program at follow-up could explain the gender effect. Moreover, in their review of the literature Pössel *et al.* [42] could establish that there are no associations of type, content or length of program, or age of participants with gender effects. There are many other aspects besides gender that may moderate the effect of a program (e.g., trainer or student variables like motivation or sociodemographic background, relationship between trainer and students, specific treatment elements like cognitive or social components) that are not analyzed in the present work.

Taking together, there is some empirical evidence for the existence of gender effects in prevention favoring female participants. Consequently, it is necessary to design studies that consider the differences in effects on female and male adolescents and to either analyze their data separately or control for a possible gender effect.

### 1.4. Hypotheses

Based on our review of the relevant literature and the positive effects of the prevention program in several studies (see below), we expected to find that adolescents benefit from participation in psychologist- and teacher-led groups but that the benefits would be greater for adolescents in psychologist-led groups. In other words, we hypothesized that the to-be-expected increase in depressive symptoms in adolescents would be significantly reduced in adolescents participating in the prevention program, with a significantly larger effect in adolescents participating in the psychologist-led condition compared to their peers in the teacher-led condition. Further, based on the literature to gender effects, we hypothesized that girls would be more likely to benefit from the prevention program than boys.

## 2. Experimental Section

### 2.1. Participants

A total of 646 adolescents attending the eighth grade participated in this study, which was conducted in 27 secondary schools in the southwest of Germany. Forty-nine principals of secondary schools were contacted, of which 26 consented for their school to take part in the study, deciding themselves if they wanted to participate in the program or take part as a control group. Our data comparing the students of the different groups did not show any differences in age, sex and baseline of the outcome measurements, only respective nationality the control group had a significant greater ratio of German students distinguished from students with a migration background.

**Figure 1:**
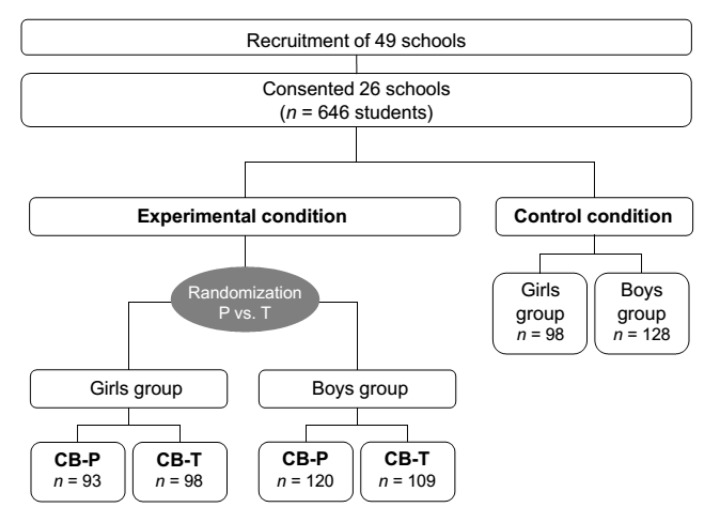
Flowchart. CB-P = psychologist-led intervention condition. CB-T = teacher-led intervention condition.

Taken together 87.5% of the students who took part completed all assessments at the four measurement times. Although we do not have data on the students’ socioeconomic status, the schools were located in economically diverse regions of the area, making it likely that the study included a broad range of social classes. In the German school system, cohorts of up to 30 students take all of their secondary school courses together. The 34 cohorts in the study were assigned to their chosen condition: to a no-intervention control condition or to a school-based universal cognitive-behavioral prevention program. The latter group was divided by gender and the two gender homogenous groups for each cohort were randomly assigned to the two experimental conditions by throwing a coin: one group to the program facilitated by psychologists (CB-P), the other to the identical program facilitated by teachers (CB-T). The students and their parents were told that they participated in a program to improve life skills and the coping with difficult situations. The term “depression” was not used to avoid stigmatization. The CB-P group consisted of 213 students, with 43.7% female; the CB-T condition consisted of 207 students, with 47.3% female, and the control condition consisted of 226 students, with 43.4% being female. For a detailed description, see the flowchart in Figure 1.

The average age of girls in the CB-P condition was 14.16 (SD = 0.97). The average age of girls in the CB-T condition (14.01, SD = 0.88) was not statistically significantly different from that (*b* = −0.18, *p* = 0.31), and neither was the average age in the control condition (13.91, SD = 0.85; *b* = −0.29, *p* = 0.10). Similarly, the average age of boys in the CB-P condition was 13.93 (SD = 0.68). The average age of boys in the CB-T condition (14.08, SD = 0.80) was not statistically significantly different from that (*b* = 0.17, *p* = 0.22), and neither was the average age in the control condition (13.92, SD = 0.83; *b* = 0.006, *p* = 0.96).

### 2.2. Measure

*Center for Epidemiological Studies—Depression Scale (CES-D).* Radloff [43] developed the Center for Epidemiological Studies—Depression Scale (CES-D) as a quickly administered, economic screening instrument to measure current depressive symptoms based on self-reports. The CES-D has been repeatedly applied to youths (e.g., [44]). The CES-D consists of 20 items, e.g., “During the past week, there were things that upset me that usually do not upset me.” On a four-point scale ranging from 0 to 3, respondents rate the frequency of symptoms, with higher numbers indicating higher frequency of occurrence. Item values are summed, creating a range from 0 to 60. In our sample, internal consistency was α = 0.86 − 0.90 at the different points of measurement. By transferring the normative data from the study of Meyer and Hautzinger [45] on the epidemiological data which reports 10% of the adolescents as “clinically depressed” and further 50% as “subclinically depressed” [46], the following cut scores result: for girls: ≥31 = “clinically depressed”; 14–30 = “subclinically depressed”; <14 = “no depressive symptoms”; for boys: ≥23 = “clinically depressed”; 11–22 = “subclinically depressed”; <11 = “no depressive symptoms”. In addition, we collected additional measures (e.g., Strengths and Difficulties Questionnaire [47,48]), but those do not allow us to measure the effects of the programs on depressive symptoms. Findings using those measures will be discussed in a later article.

### 2.3. The Cognitive-Behavioral Depression Prevention Program: LARS & LISA

The manualized school-based prevention program, LARS & LISA, was originally developed in Germany for eight graders using two psychologists as trainers [12]. The LARS & LISA intervention is based on the social information processing (SIP) model [49] and uses various methods from CB [50]. After two sessions devoted to the forming of the group and the developing of students` motivation to participate, the cognitive and social components of the social information processing model are targeted as follows: (a) four cognitive sessions focus on understanding the relations among cognitions, emotions, and behaviors and teach how to identify and challenge negative cognitions; and (b) four social sessions train participants in assertiveness and social competence skills (for a description of the links between SIP and LARS&LISA, see [35]). Two adolescent coping role-models (Lars and Lisa) accompany the students through all the topics, showing how to cope with difficult situations and change dysfunctional thoughts and behavior, appearing in many exercises and films with examples throughout the program. Implemented techniques involve role play, transfer to everyday life, positive reinforcement, *etc.*

In the current study, we adapted the LARS & LISA prevention program in order to be applicable for one trainer (psychologists or teachers) in a vocational track school (in Germany: “Hauptschule”; [51]). In terms of session contents, the adapted version is identical to the original version; however, they vary in the degree of complexity of language and difficulty of tasks according to the academic level of the target populations as LARS & LISA was intended for higher vocational track students and this adapted version was to be used with lower vocational track students of the German school system. Adaptions for this study included simplification of verbalization and work sheets, and production of educational films with peers acting as coping models (Lars and Lisa) in social situations as there were no longer two group leaders that could act as role models.

LARS & LISA is delivered to students of the 8th grade or higher (≥13 years of age) once a week over a 10-week period during regular school hours. One session is composed of two 45-min class periods for a total of 1.5-h per session. The program is administered to students in same-gender groups because adolescents may be hesitant to portray themselves authentically in front of peers of the other gender. In fact, for boys, research has shown that gender-homogeneous groups can create contexts in which they can share feelings and emotions without embarrassment [52] and can be less distracted and more open and responsive without fear of compromising their “laddish” image in front of girls [53]. For a detailed description of program contents and description see Wahl *et al.* [51].

The prevention program LARS & LISA [12] has been extensively evaluated with psychologists as group leaders in Germany (e.g., [18,34,54]), the U.S. [42], and Colombia [55]. LARS & LISA has shown positive effects on the participants’ social network [56] and symptoms of depression [18,34,54,57] compared to teaching-as-usual as well as compared to a non-specific but structurally equivalent prevention program [42] and a prevention program based on Pennebaker’s paradigm of expressive writing [58]. Teachers have shown sufficiently high quality of program delivery, adherence, and program acceptance (for details see [20]).

### 2.4. Design and Procedure

Letters were sent to the principals of 49 secondary schools in the southwest of Germany asking for their school’s participation in this project. The principals of 23 of the 49 schools refused to participate due to concerns about the potential loss of teaching time in regard of upcoming graduation exams. A written description of the study and a consent form were sent to parents of eighth-grade students in the remaining 26 schools. Additionally, information sessions were offered to teachers and parents. All but one student chose to take part in the program. This very high participation rate is typical for universal prevention programs in a school system like Germany’s [18,35,54] that has a school-cohort system in which a class of students become a cohort within the school, taking all their secondary school courses together. Students commonly have most of their friends in their school cohort and are thus highly motivated to participate in the same activities, independent of the context of these activities. This, obviously, holds true for participation in prevention programs.

Most participating schools had only one eighth-grade cohort, so it was not possible to recruit psychologist-led, teacher-led, and control conditions in the same school. As a consequence, random assignment to control or either of the two intervention conditions was realized on basis of schools and not on classes within schools. Students within one school were consequently either control or intervention condition. In the intervention schools, the cohort was divided into gender-homogeneous groups. Boys and girls were then randomly assigned either to teacher- or psychologist-led intervention condition. This leads to gender-homogeneous groups with one gender led by a psychologist and the other gender by a teacher.

Assessments were conducted in group sessions one week before the intervention (baseline), one week after the intervention (post-intervention), and at 6 and 12 months post intervention. The study was approved by the university’s ethic board.

A total of 34 cohorts participated. The 22 cohorts in the intervention conditions were split in 44 same-gender groups. In the control condition standard school curriculum was delivered to co-ed groups of students. Contrary to previous studies evaluating LARS & LISA, each of the 22 CB-P and 22 CB-T conditions were led by only one group leader. Each group was being led by a different teacher or psychologist. Training for both psychologists and teachers was provided in two steps. First, psychologists and teachers were separately trained in a 2-day workshop by two of the authors (MW, MP). Knowledge in cognitive-behavioral theory, intervention methods, and social competence training as well as understanding of depression and awareness of the negative impact of depression on the further life of students was the main focus on the first day of the teacher training. On the second day of the teacher training was similar to the second day of psychologist training (see below). Key aspect on the first day of training for the psychologists was a summary of pedagogical background such as prevention of classroom interruptions, managing a group of teenagers, lesson flow, and guidelines how to handle difficult group situations. This was trained in practical exercises using role-play. The second day of the training for both psychologists and teachers was devoted to the actual implementation of the program. First the aim of each program session was discussed in detail, and then the practical transfer into the classroom situation was taught using role play and personal examples to enhance the practical competence of the group leaders.

During the intervention, each session was videotaped with a focus on psychologists’ and teachers’ behaviors. Recordings were rated by independent, blind clinicians to ensure protocol adherence, and they were used for biweekly 1.5 h, obligatory supervision by two of the authors (MW, MP). The clinician rated whether the planned content of the session were implemented (fully, partial, not). Supervision was held separately for the psychologists and teachers. The aims of the supervision were the maintenance of adherence to the program manual, improvement of group leaders’ implementation of the program and activation the group leaders’ resources. In addition, group leaders could raise topics that seemed important to them, especially difficult classroom-situations as well as open issues and questions concerning the next sessions. Finally, the supervisors, having watched the videotape-recorded sessions, discussed all matters which could be improved.

### 2.5. Data Analysis

When groups of students (*i.e.*, gender-homogeneous groups from the same cohort) are randomly assigned instead of individuals, the observations are not independent, an assumption that traditional analyses like Ordinary Least Squares (OLS) regression make. Students in the same group interact and are more similar to one another than to students in another group, thus leading to potential intercorrelation of variables [59,60]. Therefore, to investigate the effects of the three conditions, we conducted a series of hierarchical linear model (HLM) analyses using HLM version 7.0 [61]. In the cross-sectional analyses examining differences in change-scores or in individual time points, we analyzed two-level models with students nested within group. In the longitudinal analyses examining differences in the change from post-intervention to 12-month follow-up, observations were nested within students, who were nested within group. The dependent variable of interest was depression score, which was measured at baseline, post-intervention, 6-month follow-up, and 12-month follow-up.

All analyses were conducted separately for girls and boys. This was done for two reasons. First, prior research [28,35,62] found that the shape of the change in depression over time for students receiving a similar intervention was not similar for boys and girls. Second, the boys and girls exhibited heterogeneity. Due to the number of groups in the study, we did not have enough power at the group level to examine effects of the three conditions, the gender of the students in the group (a group-level variable given groups were gender-homogeneous), and an interaction between these two variables, as well as model the heterogeneity of variances due to including both genders.

Based on graphs of individual growth, preliminary analyses, prior research, and theory, we found that change in depression from baseline to 12-month follow-up was not linear. As expected, for both genders, the CB-P condition did not have statistically significant changes from pretest to posttest (girls: *b* = −1.54, *p* = 0.26; boys: *b* = −0.28, *p* = 0.81). Furthermore, there was not a statistically significant difference from the CB-P condition in the change from pretest to posttest for the control condition (girls: *b* = −0.12, *p* = 0.95, boys: *b* = 0.81, *p* = 0.62) or for the CB-T condition (girls: *b =*−1.22, *p* = 0.51; boys: *b =* −0.96, *p* = 0.58). Because students in the three conditions reported statistically equivalent depression levels at baseline, it makes sense that at post-intervention, the students in the control condition did not report statistically significantly different depression levels from the students in the CB-P condition (girls: *b* = 1.87, *p* = 0.30; boys: *b* = 1.42, *p* = 0.18) and neither did the students in the CB-T condition (girls: *b* = 2.50, *p* = 0.17; boys: *b* = −0.18, *p* = 0.87). Given the intervention was taking place throughout the time from baseline to post-intervention, there were no effects on depression scores. We would expect any effects to manifest *after* the intervention took place. Therefore, in examining change over time, we examined the linear slope for post-intervention (time = 0 months), 6-month follow-up (time = 6 months), and 12-month follow-up (time = 12 months) and differences in that monthly growth for the three conditions, with the CB-P condition as the reference condition. As follow-up analyses, we examined differences in the conditions at each time point using cross-sectional data.

## 3. Results and Discussion

### 3.1. Baseline Comparisons

To examine baseline comparisons, we conducted cross-sectional analyses. These analyses were conducted using HLM with students nested within group and with baseline scores as the outcome measure. At baseline, students in the control condition did not report statistically significantly different levels of depression from students in the CB-P condition (girls: *b* = 2.30, *p* = 0.27; boys: *b* = 0.23, *p* = 0.86), and neither did the students in the CB-T condition (girls: *b* = 3.83, *p* = 0.07; boys: *b* = 0.45, *p* = 0.74). Furthermore, the depressive symptoms reported by the participants cover almost the entire scale range (observed range = 0 to 52; possible range = 0 to 60), indicating that depressive symptoms of all severity levels were represented in the sample.

Table 1 provides descriptive statistics for the three conditions, broken down by gender. Moreover, as we would expect, the correlations between baseline depression and depression at the other time points decreases over time (*r* = 0.48 with post-intervention and 0.25 with 12-month follow-up; *p* < 0.001).

### 3.2. Girls

**Figure 2:**
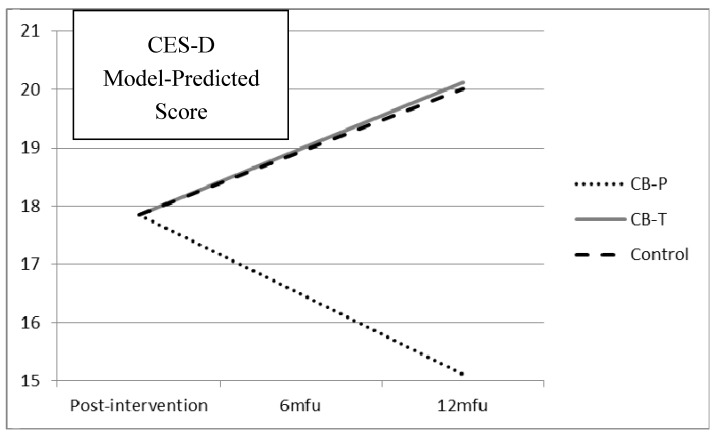
Girls’ model-predicted change in depression by condition. CB-P = psychologist-led intervention condition. CB-T = teacher-led intervention condition. mfu = month follow-up.

As shown in Table 2, girls in the CB-P condition reported a non-significant trend toward a decrease of depression scores each month from post-intervention to 12-month follow-up. On the other hand, girls in the CB-T and control conditions reported significantly more growth per month from post-intervention to 12-month follow-up. These differences in growth slopes are best illustrated using model-predicted values, which are plotted in Figure 2 (note that these are not actual means but model-predicted means at each timepoint). As shown in the figure, these differences in growth slopes result in a substantial difference between the CB-P condition and the other two conditions by 12-month follow-up. In fact, while girls in the CB-P condition reported an average *decrease* in depression by 2.74 points from post-intervention to 12-month follow-up, the girls in the CB-T condition reported an average *increase* in depression by 2.27 points and the girls in the control condition reported an average *increase* in depression by 2.16 points.

Because there were such marked differences in growth slopes for the three conditions, we conducted post-hoc cross-sectional analyses examining whether there were statistically significant differences in reported levels of depression for girls in the three conditions at 6-month follow-up and 12-month follow-up. We did this using a separate 2-level HLM with students nested within schools for 6-month and 12-month follow-up and with the depression scores at that follow-up as the outcome. We found that at 6-month follow-up, although the girls in the control condition reported statistically significantly higher levels of depression from girls in the CB-P condition (*b* = 4.26, *p* = 0.03), girls in the CB-T condition did not (*b* = 2.83, *p* = 0.14). However, at 12-month follow-up, girls in the control condition reported statistically significantly higher levels of depression from girls in the CB-P condition (*b* = 5.11, *p* = 0.02), and so did girls in the CB-T condition (*b* = 5.29, *p* = 0.02).

**Table 1:** Descriptive statistics of depressive symptoms, separated according to gender and measurement times*.*

	CB-P condition				CB-T condition				Control condition			
	Boys		Girls		Boys		Girls		Boys		Girls	
	Mean	SD	Mean	SD	Mean	SD	Mean	SD	Mean	SD	Mean	SD
CES-D at baseline	13.92	7.55	18.11	9.79	14.80	9.27	20.97	10.41	14.77	8.04	18.91	10.26
	(*n* = 120)		(*n* = 93)		(*n* = 109)		(*n* = 98)		(*n* = 128)		(*n* = 98)	
CES-D at post	13.25	6.94	17.15	11.56	13.27	7.22	17.83	10.05	14.93	7.86	18.05	10.90
	(*n* = 118)		(*n* = 88)		(*n* = 104)		(*n* = 94)		(*n* = 124)		(*n* = 96)	
CES-D at 6-mfu	14.45	8.86	15.84	11.82	15.35	10.83	18.67	10.29	14.67	8.94	20.24	11.26
	(*n* = 114)		(*n* = 79)		(*n* = 95)		(*n* = 87)		(*n* = 120)		(*n* = 90)	
CES-D at 12-mfu	16.45	9.20	14.55	10.34	13.85	9.38	19.86	11.19	15.18	9.68	19.29	10.15
	(*n* = 102)		(*n* = 64)		(*n* = 79)		(*n* = 76)		(*n* = 107)		(*n* = 77)	

Note. CES-D = Center for Epidemiological Studies—Depression Scale; mfu = month follow-up, CB-T = teacher-led intervention condition, CB-P = psychologist-led intervention condition.

**Table 2:** Fixed effects examining differences by condition in monthly change in depression from post-intervention to 12 month follow-up.

Fixed effect	Girls			Boys		
	Coefficient (SE)	*t* (df)	*p*	Coefficient (SE)	t (df)	*p*
INTRCPT3, γ_000_	17.85 (0.68)	26.30 (33)	<0.001	13.90 (0.44)	31.48 (33)	<0.001
INTRCPT3, γ_100_	−0.23 (0.11)	−2.03 (31)	0.051	0.21 (0.08)	2.51 (31)	0.018
CB-T, γ_101_	0.42 (0.14)	2.93 (31)	0.006	*−*0.10 (0.11)	*−*0.83 (31)	0.413
CONTROL, γ_102_	0.41 (0.14)	2.88 (31)	0.007	*−*0.12 (0.11)	*−*1.11 (31)	0.275

Note. The reference group was the psychologist-led condition (CB-P). Estimates provide the differential in monthly change for the teacher-led condition (CB-T) and the control condition.

### 3.3. Boys

As shown in Table 2, boys in the CB-P condition reported a statistically significant increase in depression scores each month from post-intervention to 12-month follow-up. However, both the CB-T and control conditions did not have statistically significant differences in that change over time. These results are supported with post-hoc cross-sectional analyses examining whether there were statistically significant differences in reported levels of depression for boys in the three conditions at 6-month follow-up and 12-month follow-up. At 6-month follow-up, the boys in the control condition did not report statistically significantly different levels of depression from the CB-P (*b* = 0.02, *p* = 0.99), and neither did boys in the CB-T condition (*b* = 0.69, *p* = 0.63). Similarly, at 12-month follow-up, the boys in the control condition did not report statistically significantly different levels of depression from the CB-P (*b* = −1.27, *p* = 0.34), and neither did boys in the CB-T condition (*b* = −2.61, *p* = 0.08).

### 3.4. Discussion

The aim of the present study was to evaluate a well-established, school-based intervention program designed to prevent the increase and development of depressive symptoms in adolescents under real-school-life conditions and to compare the effects when led by a psychologist or by a teacher and the trajectory of a control group. On the basis of previous results, we expected to find prevention effects on depressive symptoms in both the psychologist-led and the teacher-led intervention compared to the control condition (standard curriculum). We also expected to find significantly stronger effects for psychologists compared to teachers, and we expected girls to profit more from participating in the program than boys.

Consistent with previous research (e.g., [35]), the change in depressive symptoms differed for girls and boys. Boys did not profit from the program in either the psychologist-led or the teacher-led intervention condition. In the psychologist-led condition the depressive symptoms in boys even increased from post-intervention to 12-month follow-up. Yet, as post-hoc cross-sectional analyses revealed, there were not statistically significant differences between boys in the CB-P condition and in the other two conditions—either at 6-month or at 12-month follow-up.

As expected, girls benefitted most from LARS & LISA, in particular in the psychologist-led condition. Main analyses showed a nonsignificant decrease of depressive symptoms over time in the psychologist-led condition, while depressive symptoms in the teacher-led condition and the control condition significantly increased over time up to one year after the intervention. Post-hoc cross-sectional analyses revealed significant differences between girls in different conditions: At 6-month follow-up, girls in the psychologist-led condition showed significant lower levels of depressive symptoms than girls in the control condition, while there was no significant difference between girls in psychologist-led *vs.* teacher-led condition. At 12-month follow-up, however, significantly lower levels were found for girls in the psychologist-led condition in comparison to girls in control and teacher-led condition.

These positive long-term effects for girls of the prevention program LARS & LISA implemented by psychologists is in line with previous research results (e.g., [7,26]). Also consistent with previous studies using LARS & LISA [35] and other prevention programs [33] is the fact that boys did not benefit from the prevention program, independent of the qualification of the group leader.

We did not find positive intervention effects for girls or boys on depressive symptoms in the teacher-led condition. This result is in line with a meta-analysis that found long-term positive outcome only when healthcare professionals delivered the prevention program, in contrast to classroom teachers [14]. It is also consistent with the results of a recent Dutch study [63], in which a similar sample of students of low income areas did not profit from a teacher-led depression prevention program. Researchers [14,16,21,24] have speculated that a lack of specific training, a lack of general knowledge in psychology, or no (repeated) experience in delivering the program might be responsible for these differences between psychologists and teachers. We tried to control for some of these influencing factors by intensive training and supervision for teachers with emphasis on theoretical background, cognitive and learning theory, social skills training, as well as handling of students and their interactions, more positive reinforcement, biweekly feedback, and reviewing videotapes of sessions. However, it was our impression that psychologists used supervision and feedback even more intensively than the teachers. In terms of accumulated experience in program delivery, our study psychologists were not more experienced than the study teachers. Our study teacher’s quality of program delivery, adherence to program, and program acceptance was satisfactory [20].

Besides these moderating group leader variables, other factors could have influenced the outcome, including: group leaders’ social competence (general skills), motivation, attitude toward and acceptance of the program, and more intensive and individualised supervision [10,16,25,64,65]. Future research should address these questions in order to determine which factors influence the effectiveness of prevention programs, especially when delivered by teachers.

The observed gender effect cannot be explained by regression to the mean as suggested by Stice *et al.* [14] because in our study boys not only changed less but also showed a tendency in the opposite direction. In addition, dominance of the boys in co-ed groups, a suggested explanation of gender effects [26], did not play a role here because we trained only gender-homogenous groups.

Because we found a gender effect only in the psychologist-led condition, the most likely explanation could be disciplinary problems within boy groups. Psychologist are less experienced in handling difficult, dominating, and demanding boys with divergent cultural background, as we find them in the German vocational track school system. This type of school is the track of the tripartite school system in Germany, with a large portion of challenging students (incl. immigrants, students with learning difficulties, and conduct problems [66,67]). Especially the external psychologists, who have less experience with being in charge of this kind of group, no prior systems of rules, and no disciplinary power over grades, might have trouble handling difficult disciplinary situations and covering the prevention program as well. This assumption is supported by the fact that in this study psychologists assessed the disruptive behavior of boys significantly higher than teachers did [68]. We tried to compensate for the lack of pedagogical and disciplinary knowledge in the psychologists’ condition by focussing on these topics in the training workshop training and the supervision, but perhaps these skills cannot be taught in such a short time [69]. However, as mentioned above, Pössel *et al.* tested whether conduct behavior could explain the gender effect they found and found that after controlling for conduct behavior, the gender effect in which girls but not boys benefited from participating in a prevention program remained [35].

The result that boys in the psychologist-lead group not only did not exhibit an improvement in depressive symptoms but even deteriorated could be an iatrogenic effect. In the study of Kindt, Kleinjan, Janssen und Scholte [63] both genders had a similar iatrogenic effect in the secondary outcome of clinical depressive symptoms. The authors presume that the program led some of the students to focus more on their deficits and negative thoughts instead of focusing more on their resources and alternatives. A possible explanation is that the program led them to focus more on their deficits and negative thoughts instead to focus more on their resources and alternatives. Another explanation is provided by Gollwitzer, Hartmann, and Pfetsch [70], who also found deterioration effects in boys participating in their program and concluded that boys answered the questionnaire reluctantly. However, if this explanation would be true for the present study, it is unclear why this response behavior would appear in the psychologist-led groups on a larger scale than in the teacher-led groups as the students responded to the questionnaire confidentially and the group facilitator would not be accessing this data. Another possible explanation for the gender effect could be that girls are more motivated to participate, engage more actively in the exercises, and are more interested to learn the content of the program [41]. This might be related not only to their less disruptive behavior and generally more social responsiveness but also to the fact that they experience more depressive symptoms and try to use the program skills to overcome these symptoms. It may be that boys are less interested in the topics of the program because they are less burdened by symptoms [32]. Finally, it is possible that the content of the program focusing on emotions and assertive instead of aggressive behavior runs counter to the role model of adolescent men [71] and therefore is less attractive for the boys. Correspondingly, in our study, external observer rated the girls as more actively engaged than the boys, at the same time the girls showed a higher acceptance of the program than the boys [68]. Based on the above, one could expect that girls know more about the content of a prevention program and that controlling for knowledge would diminish the gender effect; however, Pössel *et al.* [35] tested this hypothesis and found only partial support for this hypothesis.

For the interpretation of the present results, it is important to consider several limitations. First, missing data and absenteeism of students are a constant problem. Some students do not take part at all in the assessment and others missed school and therefore the assessment at one point or another. However, in our study the rate of students who took part at all assessments was very high. This high rate can be explained by the German school cohort system where all students of one cohort take classes together and therefore are motivated to participate in same activities. This is consistent with Masia-Warner *et al.*’s [17] recommendation that schools are the single location where the majority can be reached. Other factors likely contributing to the high participation rate are that assessment as well as training sessions took place during regular school hours and the easy handling through online questionnaires. Moreover, for the longitudinal analyses, we used maximum likelihood estimation, which uses all available information rather than removing individuals with missing assessment scores.

A further limitation is the missing randomization on school level. This is a problem often seen in implementation and efficacy studies, and it could lead to a selection bias of schools and a conflation of school *versus* intervention effects. Even if in most variables the students of the control group did not differ statistically from those from the experimental group, the smaller ratio of students with migration background could mean that they were the “easier classes”. If that were the case, it could have a positive influence on the expected development of depressive symptoms, so a bias against the positive effects of the program is to be expected. In addition the two groups might differ in other influential variables which were not surveyed. Although the lack of school-level randomization lessens the statistical validity, it does contribute to the ecological validity, as in real life schools and teachers decide themselves if they want to implement a program and this motivation will influence the results of their endeavors [16].

Another limitation is that the students in the control condition took part in their regular school curriculum, which is, in fact, a common procedure in most prevention studies. Including a more active control condition in future research is necessary to separate program specific effects from non-specific factors like attention and support by an “enthusiastic and charismatic” group leader [24]. However, it does not seem plausible that only girls in psychologist-led groups were influenced by attention processes in our study.

The exclusive use of self-report measures is another limitation of our study. Although previous studies have shown that adolescents are a reliable and valid source of information for depressive symptoms [72,73], social desirability might have caused our adolescents sample to answer the questionnaire in a desired manner. Self-report measures might lead to social desirability differentially depending on the qualification of the group leader. Teachers can be expected to have a greater and sustainable impact on students’ life in comparison to psychologists as group leaders because they will continue to work with the students in schools after program delivery and also give academic grades to them. In addition, as the students possibly will not have contact with the program leading psychologists again after program delivery, it is likely that they care less about them and are less likely influenced by social desirability. Both scenarios should lead to more social desirable answers from students in the teacher-led condition compared to the psychologist-led condition. Thus, one would expect to find better “effects” on students’ depression scores by the teacher-led prevention program than by the psychologist-led program.

Finally, we did not measure whether the skills trained in LARS & LISA were implemented by the students in their daily life. Addressing these outcomes by behavioral observation measures and modern electronic devices should be part of future research.

## 4. Conclusions

Summarized, positive intervention effects were found on the change in girls’ depressive symptoms up to 12 months after program delivery when the program was implemented by psychologists. No such effects were found on boys or when program was delivered by teachers. The prevention program can successfully be implemented for girls by psychologists. Future research needs to address the question of what makes psychologists compared to teachers more successful in preventing depression in adolescents and what possible mechanisms may explain why girls benefit more from such a prevention program than boys. Research should focus on factors concerning group leaders’ social competence (general skills), motivation, attitude toward and acceptance of the program, and intensity and individualization of supervision.
